# Confluent soft nodules on buttocks

**DOI:** 10.1016/j.jdcr.2025.01.029

**Published:** 2025-02-22

**Authors:** Awa Bakayoko, Chiamaka Ohanenye, Jason B. Lee, Shaan Patel, Sylvia Hsu

**Affiliations:** a Department of Dermatology, Temple University Lewis Katz School of Medicine, Philadelphia, Pennsylvania; b Department of Dermatology and Cutaneous Biology, Jefferson University Hospital at Sidney Kimmel Medical College, Philadelphia, Pennsylvania

**Keywords:** cosmetic procedure, silicone granuloma

## Case presentation

**Fig 1:**
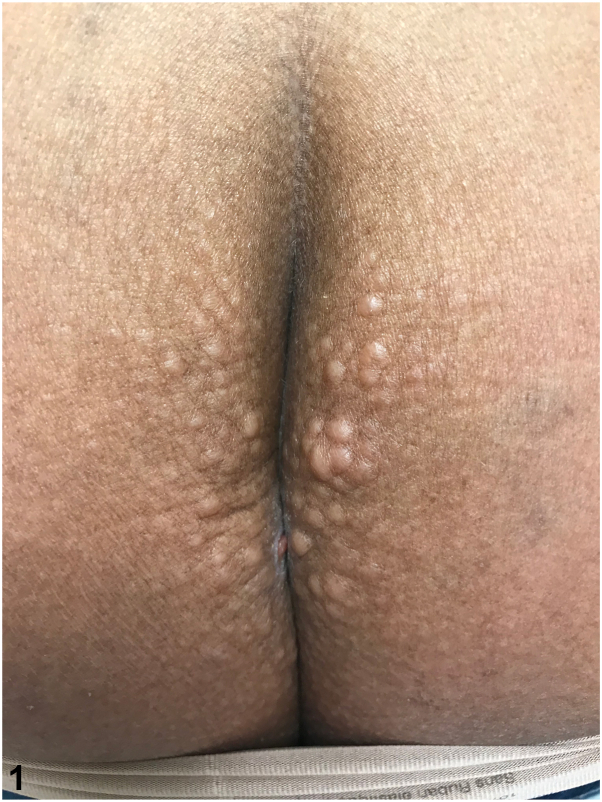

**Fig 2:**
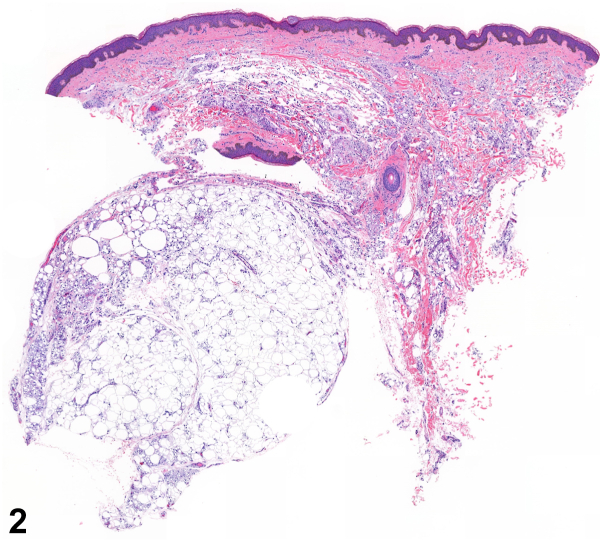

**Fig 3:**
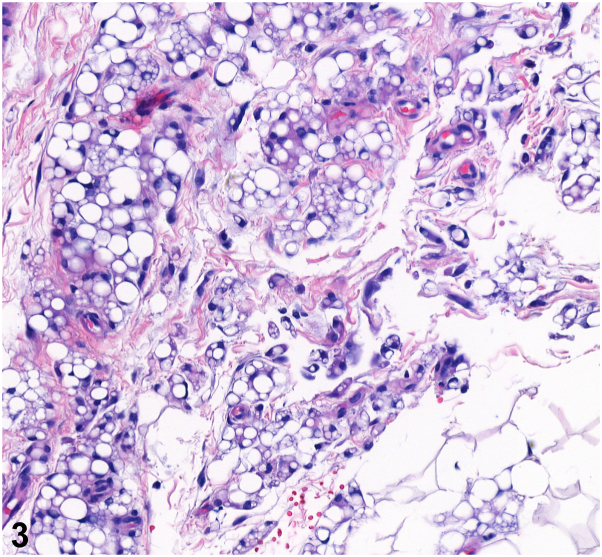

A 45-year-old Hispanic woman presented with asymptomatic nodules on her buttocks. She denied any trauma or application of new topical agents to the area. On physical examination, the patient is noted to have confluent 1-2 cm soft, skin-colored nodules on bilateral medial buttocks (Fig 1). A 4-mm punch biopsy of the right medial buttock was performed (Figs 2 and 3).

**Question 1: Based on the physical examination and dermatopathology, what is your diagnosis?**

A. Elephantiasis nostras verruciformis
B. Neurofibromas
C. Pseudoverrucous papules
D. Shagreen patch
E. Silicone granuloma

**Answers:**

A. Elephantiasis nostras verruciformis – Incorrect. Elephantiasis nostras verruciformis occurs in the setting of chronic lymphedema, resulting in hypertrophic cutaneous changes in gravity-dependent areas. Histopathology would show dilated lymphatic channels and epidermal hyperplasia, not lobules of fat cells with multinucleated giant cells.
B. Neurofibromas – Incorrect. Neurofibromas are a type of cutaneous nerve tumors that present in patients with neurofibromatosis. Neurofibromas can present as soft nodules; however, histopathology would show Schwann cells, fibroblasts, and perineural cells.
C. Pseudoverrucous papules – Incorrect. Pseudoverrucous papules are commonly described in patients with colostomies, urostomies, or chronic fecal incontinence, as these areas tend to entrap moisture and heat, which can irritate the skin. Histopathology would show epidermal hyperplasia and hyperkeratosis.
D. Shagreen patch – Incorrect. Shagreen patches are firm yellow/pink patches or plaques on the lumbar region and are associated with tuberous sclerosis. This patient does not report of a history of tuberous sclerosis or of findings consistent with tuberous sclerosis, such as epilepsy, developmental delay, ash-leaf spots (hypomelanotic macules), or angiofibromas.
E. Silicone granuloma – Correct. Figs 2 and 3 depict numerous small nucleated vacuolated spaces throughout the dermis and subcutis and eccentrically nucleated vacuolated histiocytes with larger variably-sized fat microcysts, consistent with a diagnosis of silicone granuloma. This, in addition to the location of the nodules on the patient, should raise suspicion for a granulomatous reaction secondary to silicone injection.

**Question 2: What laboratory finding can be associated with the diagnosis?**

A. Hypokalemia
B. Hypercalcemia
C. Hypernatremia
D. Hyperglycemia
E. Hypophosphatemia

**Answers:**

A. Hypokalemia – Incorrect. Derangements in potassium levels are not associated with silicone granulomatous reactions.
B. Hypercalcemia – Correct. Granulomatous reactions are associated with hypercalcemia due to the 1 α-hydroxylase activity of multinucleated giant cells. However, many patients may not present with signs/symptoms or laboratory values concerning for hypercalcemia.^1^
C. Hypernatremia – Incorrect. Derangements in sodium levels are not associated with silicone granulomatous reactions.
D. Hyperglycemia – Incorrect. Derangements in glucose levels are not associated with silicone granulomatous reactions.
E. Hypophosphatemia – Incorrect. Granulomatous reactions can cause derangements in calcium, which can affect phosphate homeostasis. However, high levels of calcium caused by granulomatous reactions would lead to decreased release of parathyroid hormone leading to hyperphosphatemia.

**Question 3: Of the following, which is the most common complication that can be seen with this diagnosis?**

A. Pneumonitis
B. Hepatitis
C. Amaurosis fugax
D. Heart failure
E. Lymphadenopathy

**Answers**:

A. Pneumonitis – Incorrect. Although silicone-induced pneumonitis can be observed years following injection, this is not the most common complication seen in these patients.^2^^,^^3^
B. Hepatitis – Incorrect. Hepatitis has not been observed in relation to the use of silicone injections.
C. Amaurosis fugax – Incorrect. Literature review shows few case reports depicting this symptom after injection of silicone oil-induced occlusion of the posterior ciliary artery.^3^^,^^4^
D. Heart failure – Incorrect. Heart failure has not been observed in relation to the use of silicone injections.
E. Lymphadenopathy – Correct. Years after injection, leakage and migration of silicone particles can occur, giving rise to a host of complications. The most common complications seen with migration include silicone granulomas and silicone lymphadenopathy.^3^

## Conflicts of interest

None disclosed.
